# Structural Mechanism Shows How Transferrin Receptor Binds Multiple Ligands and Sheds Light on a Hereditary Iron Disease

**DOI:** 10.1371/journal.pbio.0000029

**Published:** 2003-12-22

Iron is an essential nutrient for sustaining life-forms as diverse as plankton and humans. But too much iron, or too little, can spell trouble. Mammalian cells maintain the proper balance partly with the help of a specialized cell surface protein called the transferrin receptor (TfR). TfRs bind to the iron-carrying transferrin protein (Fe-Tf) and escort their cargo to the cell's interior. (To learn more about iron metabolism, see the primer by Tracey A. Rouault in this issue [DOI: 10.3171/journal.pbio.0000079].) This receptor also binds the hereditary hemochromatosis protein (HFE), which is mutated in individuals who have the common iron-overload disorder hereditary hemochromatosis. While the molecular pathway that mediates cellular intake of iron through the TfR is known, it was not clear just how TfR assists in iron release to the cell and how it binds HFE and transferrin. By introducing multiple mutations in human TfRs, Pamela Bjorkman and colleagues identified functional binding sites for transferrin in both its iron-loaded and iron-free (apo-Tf) forms and for HFE. From these data, the researchers mapped out a scenario of the dynamic interactions between receptor and ligands (the bound molecule) and worked out a structure-based model for the mechanism of TfR-assisted iron release from Fe-Tf.

Bjorkman's lab, which had previously solved the structures of both HFE and HFE bound to the TfR, used their structural information to investigate how the proteins interact, which amino acid residues are required for binding, whether the two ligands bind differently to the receptor, and how HFE binding affects transferrin binding. They found that Fe-Tf and HFE occupy the same or an overlapping site on the receptor, but since transferrin is much larger than the HFE protein, it appeared that transferrin could also interact with other parts of TfR. And it remained to be seen whether TfR discriminated between the iron-loaded and iron-free states of transferrin.

In this study, Bjorkman and colleagues expanded their library of TfR mutants to clarify the transferrin binding signature on TfR and to see how the TfR mutations affect the way apo-Tf and Fe-Tf interact with the receptor. They characterized the binding affinities of 30 TfR mutants to HFE and Fe-Tf and to apo-Tf, and they report that mutations in 11 of the TfR residues interfere with either one or both forms of transferrin. Four of these residues are essential for transferrin binding and are conserved in all known TfR DNA sequences. Since residues that didn't have much impact are not conserved, the scientists say the results are likely to describe transferrin–TfR interactions for other species as well.

As expected, the most critical residues required for transferrin binding fall within the receptor's helical domain and have significant physical overlap with residues required for HFE binding; though some residues that are required for apo-Tf binding do not affect Fe-Tf binding. Bjorkman et al. also identify additional residues in another domain on TfR (called the protease-like domain) that support Fe-Tf but not apo-Tf binding, confirming that the receptor binding footprints of the two metal-binding states of transferrin are indeed different. With a structural model showing *where* Fe-Tf and apo-Tf bind to the receptor, they could evaluate *how* they bind and thus explain how the receptor mediates iron release.

By suggesting a mechanism through which TfR binding regulates iron release, this structural model of the transferrin–TfR complex will bolster efforts to elucidate the molecular details of this process. Confirmation that transferrin and HFE do indeed compete for docking privileges reveals a possible role for HFE in maintaining iron homeostasis and will provide valuable insights into the dysregulation that leads to the warehousing of iron and resulting tissue and organ damage associated with hemochromatosis.

**Figure:**
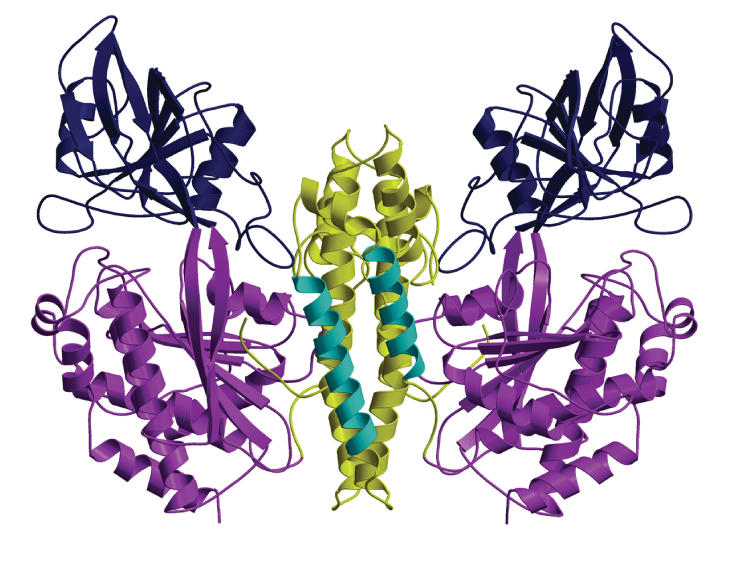
Ribbon diagram of transferrin receptor homodimer

